# Skull Biomechanics and Simplified Cephalometric Lines for the Estimation of Muscular Lines of Action

**DOI:** 10.3390/jpm13111569

**Published:** 2023-11-01

**Authors:** Claudia Dolci, Niccolò Cenzato, Cinzia Maspero, Lucia Giannini, Shahnawaz Khijmatgar, Gianna Dipalma, Gianluca Martino Tartaglia, Francesco Inchingolo

**Affiliations:** 1 Department of Biomedical Sciences for Health, Università Degli Studi di Milano, 20133 Milan, Italy; 2 Fondazione IRCCS Ca’ Granda, Ospedale Maggiore Policlinico, 20122 Milan, Italy; niccolo.cenzato@unimi.it (N.C.);; 3 Department of Biomedical, Surgical and Dental Sciences, School of Dentistry, Università Degli Studi di Milano, 20100 Milan, Italy; 4 Department of Interdisciplinary Medicine, Università Degli Studi di Bari “Aldo Moro”, 70124 Bari, Italy; giannadipalma@tiscali.it (G.D.);

**Keywords:** cephalometric analysis, skeletal classes, muscular lines

## Abstract

Our study introduces a novel cephalometric analysis aimed at facilitating biomechanical simulations by elucidating the intricate relationship between craniofacial morphology and the size and inclination of the masseter muscle (MM) while incorporating muscle values. Our study analyzes the line of action of the MM drawn between the Gonion (Go) and Orbital (Or) points concerning dental and skeletal references (occlusal and Frankfort planes). A total of 510 pre-treatment lateral cephalometric tracings (217 males, 293 females, aged 6–50 years) and lateral Bolton standard tracings were examined. The key parameters investigated include (a) skeletal-cutaneous class (linear distance between projections of points A′ and B′ on the occlusal plane), (b) the angle between the perpendicular line to the occlusal plane and the Go-Or line at the molar occlusal point, and (c) the angle between the Go-Or line and the Frankfort plane. The assessment of anterior-posterior jaw discrepancy, measured as the skeletal-cutaneous class, ranged from −14.5 to 15.5 mm. Abnormal values were identified in two adolescents, showing no gender- or age-related patterns. The angle between the MM’s line of action (Go-Or) and the normal to the occlusal plane averaged 39.3°, while the angle between Go-Or and Po-Or (Frankfort plane) averaged 41.99°. Age had an impact on these angles, with an average 3° decrease in adults and a 4° increase between ages 6 and 50. A weak relationship was observed between sagittal jaw discrepancy and the angle between Go-Or and the Frankfort plane, with about 20% of the variance explained by the anteroposterior maxillary-mandibular relationship. In conclusion, the study presents a cephalometric analysis of the relationship between craniofacial morphology and masseter muscle parameters. It finds that age influences the angles between key reference points, while the skeletal-cutaneous class does not exhibit age- or gender-specific trends. These findings can contribute to a better understanding of craniofacial biomechanics and aid in clinical orthodontic assessments and treatment planning.

## 1. Introduction

The goal of orthodontic treatment is to achieve neuromuscular balance and a stable occlusion [1]. The performance of the masticatory muscles is one of the functional variables that can be altered by orthodontic and surgical modification of the dental arch and craniofacial skeleton [2]. The assessment of the actual bite force is difficult because bite force sensors often interfere with occlusion and the relationship between electromyographic signals and bite force is nonlinear [3]. Except for relatively complex imaging techniques such as computed tomography, magnetic resonance imaging, and ultrasound which cannot be performed extensively, biomechanical models from conventional radiographs are primarily used. In these models, muscle forces and occlusal resistance are estimated from geometric variables measured against tooth and skeletal landmarks [4,5,6,7]. For this purpose, the mechanical advantage of the masticatory muscles is determined, specifically the ratio of the muscle moment arm to the occlusal force moment arm.

This ratio depends on the line of action of the muscle and the associated moment arm. The position of the occlusal force is usually defined as perpendicular to the occlusal plane at the molar or incisor midline, but there is no agreement on the direction of the lines estimating the different masticatory muscles. In particular, the superficial part of the masseter muscle (MM) is estimated by a line connecting the gonion (Go) and several cephalic landmarks: (a) the orbit (Or), (b) the intersection of the flattened process of the zygomatic bone with the frontal process, and (c) the zygomatic bone (the lowest point on the outline of the zygomatic process on the zygomatic bone) [4,5,6,8,9]. In addition, a line connecting the anterior root of the zygomatic arch with the zygomatic-temporal suture and the midpoint of Go and the anterior root, and a line drawn parallel to the anterior root-key ridge line from the midpoint of Go and the anterior root have been proposed [10,11].

This lack of congruence can be overcome by directly analyzing the muscle itself, either by actual autopsy (postmortem) or virtual autopsy (magnetic resonance or computed tomography). These articles [11,12,13,14,15,16,17,18,19,20] collectively contribute to our understanding of craniofacial biomechanics, spanning various topics and methodologies. Gionhaku’s 1989 [11] study evaluates the relationship between craniofacial form and jaw muscle function in subjects with Obstructive Sleep Apnea. Hannam’s 1989 work aims to establish a connection between craniofacial form and jaw muscle function in individuals with Obstructive Sleep Apnea [12]. Kasai’s 1994 study explores the attachment and orientation of the superficial masseter muscle in dentate and edentulous individuals [13]. Koolstra’s 1990 study examines the accuracy of estimating muscle orientation in healthy subjects using MRI [14]. Van Spronsen’s 1996 [15] work investigates the relationship between craniofacial morphology and superficial masseter muscle in dentate and edentulous subjects. Broadben BH 1975 looked at the bolton standards of dentofacial development growth [16]. Van Eijden’s 1997 [17] study compares architectural characteristics of jaw-closing and jaw-opening muscles, shedding light on their roles in force production and velocity. Prado’s 2014 [18] study highlights the significance of masticatory stress dissipation in Dentistry and the utility of Finite Element Analysis (FEA). Sharp et al. [19] in 2023 explore the role of cranial sutures in overall skull biomechanics and their importance in specific region. Watson’s PJ 2021 [20] research delves into the biomechanics of rabbit skulls during mastication, revealing insights into strain distribution These studies collectively provide valuable insights into craniofacial biomechanics, muscle architecture, and the mechanical properties of cranial sutures.

Unfortunately, both ex vivo and in vivo studies have mostly involved small samples of adults and have not shown correlation with x-ray analysis [11,12,13,14,15,16,17]. Furthermore, to our knowledge no biomechanical studies have evaluated the relationship of these hypothetical lines of action to other craniofacial structures.

The novelty of conducting this study has significant importance within the field of orthodontics as it aims to address a fundamental aspect of orthodontic treatment—achieving neuromuscular balance and a stable occlusion. By investigating the lines of action of masticatory muscles and seeking to standardize these measurements, a crucial diagnostic tool is provided that can improve the precision of orthodontic assessments. Its inclusion of a diverse sample covering a wide age range enhances the generalizability of findings and makes them applicable to various clinical scenarios, including pediatric considerations. Additionally, by exploring how factors such as sex, age, and skeletal-cutaneous class may impact muscle function, the study offers the potential to tailor treatment plans to individual patient characteristics. This research effectively bridges the gap between anatomy and orthodontics, ultimately contributing to the improvement of treatment outcomes, patient comfort, and the long-term stability of orthodontic results.

In the present study, the position of the presumed MM line of action drawn between Go and Or [6], relative to dental (occlusal plane) and skeletal (Frankfort plane) standards, was analyzed in (a) a large sample of unselected orthodontic patients of a wide age range, and (b) lateral tracings of Bolton standards (male and female average) aged 6 to 18 [16]. In addition, its relationship with sex, age, and skeletal-cutaneous class (soft tissue equivalent of the Wits appraisal, [21,22]) has also been studied.

## 2. Materials and Methods

### 2.1. Sample

Pre-treatment lateral cephalometric tracings of 510 orthodontic patients (217 males and 293 females, aged 6–50 years) were used. The patient records used in this cross-sectional study were obtained from the dental department of the Fondazione IRCCS Ca’ Granda Ospedale Maggiore Policlinico, Milan, Italy. Cephalograms were obtained by cone beam computed tomography (CBCT), as currently used in dentistry [23].

The study population included patients with the following dentoskeletal characteristics at the time of pretreatment lateral cephalometric imaging:

1. be of European (Caucasian) descent;
2. malocclusion that could be corrected by orthodontic treatment alone, as determined by a specialized orthodontist;
3. complete primary or permanent dentition (excluding third molars);
4. a maximum difference of 3 mm in the distance between each crest and maxillary point from the mid-sagittal plane in the posterior-anterior projection according to Hwang et al. [24];
5. no crossbite as reported in the patient’s records and confirmed by CBCT scan.

### 2.2. Exclusion Criteria Were as Follows

1. missing molars or bicuspids;
2. a history of orthodontic treatment;
3. altered bone metabolism;
4. skeletal asymmetry greater than 2 mm on the left and right cephalograms;
5. syndromic disorders (acquired or congenital);
6. patients requiring surgery were not evaluated.

The objective of selecting orthodontic subjects was to design a simplified model that minimizes measurement error compared to interindividual variability [25].

Patients were divided into three non-overlapping age groups: 6–10 years (children), 11–15 years (adolescents), and 16–50 years (adults), all rounded to the nearest 6 months.

Details of the technique are described by Ferrario et al. [22,26]; Bolton standard lateral tracings were digitized as previously described by Ferrario et al. [21,22].

### 2.3. Measurements

**Figure 1:**
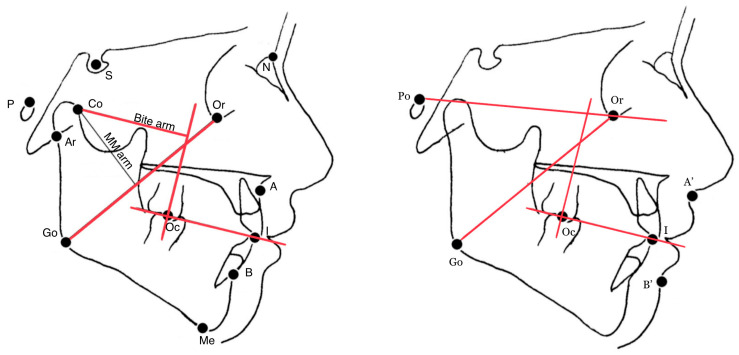
Digitized cephalometric landmarks. Po = Porion; Or = Orbital; Go = Gonion; I = inter-incisal point; Oc = occlusal point of first permanent molar; A′ = soft-tissue sub-spinal point; B′ = soft tissue supramental point; The estimated MM line of action connects Go and Or.

CBCT raw data was stored in Digital Imaging and Communications in Medicine file format (DICOM3). Lateral radiographic projections of the entire volume were reconstructed for each raw data set using iCAT Vision (Imaging Sciences International Inc., London, UK, https://ct-dent.co.uk/i-cat-vision/, accessed on 10 February 2021), according to Baldini et al. [25]. All 2D cephalograms were then traced by two expert orthodontists (NC, CM) using dedicated software (Dolphin Imaging Cephalometric and Tracing Software, V 11.9, Chatsworth CA, USA, https://www.dolphinimaging.com/product/Imaging?Subcategory_OS_Safe_Name=Ceph_Tracing, accessed on 10 February 2021). Cephalometric points on CBCT scans were first identified in one plane (axial, coronal or sagittal) and then checked in the other two and in the 3D volumetric rendering (Figure 1). Linear and angular measurements were obtained by means of computer software currently in use at our laboratory, according to Farronato et al. [23].

Among others, the following measurements were selected and analyzed:

1. skeletal-cutaneous class (soft tissue equivalent of the Wits appraisal, i.e., the linear distance (mm) between the projections of the points A′ and B′ on the bisecting occlusal plane (OP), i.e., the plane bisecting the overbite of the molar and incisor teeth [19];
2. the angle between the Go-Or line (estimated MM action line) and the perpendicular line to the bisecting occlusal plane through the molar occlusal point (Oc);
3. the angle between the Go-Or line and the Frankfort plane (Po-Or).

### 2.4. Error Evaluation Method

Intra- and inter-operator reliability of the analyzed cephalometric measurements (ANB and AFBF) has been investigated in a previous study [23]. Briefly, three independent observers with the same professional background and five years of orthodontic experience performed three cephalometric analyses at 15-day intervals. Intraclass correlation coefficient (ICC) estimates and their 95% confidence intervals for intra- and inter-rater reliability were calculated using SPSS^®^ 25.00 for Windows™ (single-measure, absolute agreement, two-way mixed effects model for each variable). Based on this, for the present calculations, two expert operators were calibrated in a training session, where the inter-examiner agreement on the tested characteristics was set to 95%.

In addition, a random sample of 30 images was retraced and re-digitized by the same investigators one month later. Each set of cephalometric landmark coordinates was normalized with respect to rotation and translation by placing the origin of the axis at the center of gravity of the coordinates and aligning the X axis with the Frankfort plane (Po-Or). Each pair of repetitions was then compared between landmarks. Repeated digitization of the same traces produced differences of less than 2 mm (average 1.2 mm), and repeated tracing of the same radiographs produced differences of less than 2.5 mm (average 1.8 mm).

A linear correlation analysis between the measured variables was performed. Significance was set at α level of 5% (i.e., *p* ≤ 0.05). Univariate (for linear variables) and bivariate (for angles) statistics were used to calculate means within sex and age groups [9].

### 2.5. Estimation of Sample Size

To estimate the line of action of MM from the lines of the simplified cephalogram, the results considered were Wits appraisals (see above). The skeletal-cutaneous class values for all patients before treatment, as measured by the lateral cephalogram, ranged from −14.5 to 15.5 mm, with no differences by sex or age. Therefore, with a significant difference *p* < 0.05, power of 0.8, mean difference in Wits values of 2.5 mm, and SD of 5.0 mm, the minimum sample size required for the study was *n* = 140 (*n* = 70 for each group). The sample size was calculated using STATA version 18.0.

## 3. Results

### 3.1. Orthodontic Sample

The assessment of anterior-posterior jaw discrepancy, measured as the linear distance between the soft tissue A′ and soft tissue B′ projections on the occlusal plane (skeletal-cutaneous class or “soft tissue” Wits, [22]), ranged from −14.5 to 15.5 mm. Abnormal values were found in two adolescents, the smallest being a 15-year-old girl and the largest being a 14-year-old boy. No specific gender- or age-related behavior was observed for this distance (Table 1), and the linear correlation coefficient with age was only 0.025 (Table 2).

The average angle between the estimated line of action (Go-Or) of the MM and the normal of the occlusal plane was 39.3°. On the other hand, the angle between the lines of Go-Or and Po-Or (Frankfort plane) averaged 41.99°, ranging from 30.4° to 53.8° (Table 1). No effect of gender was observed. The angle value decreased with increasing age, and on average was about 3° smaller in adults than in children. Conversely, the angle between the Go-Or line and the Frankfort plane increased by an average of about 4° between ages 6 and 50. In fact, the correlation analysis between age and this variable showed a correlation coefficient of r = 0.449 (Table 2), which was the largest age effect found in the present orthodontic sample. The two angles were also significantly correlated.

Although the relationship between the sagittal jaw discrepancy and the angle between the estimated MM action line and the Frankfort plane was poor, about 20% of the variance in the angle between the Go-Or line and the normal of the occlusal plane was explained by the anteroposterior relationship between the maxilla and mandible (Table 2).

Linear correlation coefficients revealed a weak relationship between age and skeletal-cutaneous class (0.025) and a somewhat stronger correlation between age and the angle between Go-Or to normal to OP (0.255) (Table 2). There was a moderate correlation between age and the angle between Go-Or to Po-Or (0.449).

Skeletal-cutaneous class = linear distance between the projections of A′ and B′ points on the occlusal plane.

Skeletal-cutaneous class (linear distance between the projections of A′ and B′ points on the occlusal plane) had a mean value of 4.2 mm, with a standard deviation of 4.0. Variations within different age groups were observed, ranging from 3.4 mm in patients aged 11–15 years to 4.8 mm in those aged 16–50 years.

### 3.2. Bolton Tracings

Overall, the mean values of the three variables measured in Bolton tracings were like those found in orthodontic patients of similar age (Table 3), but the differences were less than 1 mm (skeletal-cutaneous class) and 2.5° (angles). The correlation between age and the angle between the Go-Or line and the Frankfort plane was particularly strong (Table 4).

The study also examined Bolton tracings in a population of individuals between the ages of 6 and 18 years. The skeletal-cutaneous class showed a mean value of 3.5 mm and a standard deviation of 1.2 mm. The angle between Go-Or to normal to OP had a mean value of 38.77° and a standard deviation of 0.47°. The angle between Go-Or to Po-Or averaged 40.72° with a standard deviation of 0.55° (Table 3). Strong correlations were found between age and the angle between Go-Or to Po-Or (0.965) and the angle between Go-Or to normal to OP (0.397). A slightly weaker correlation was observed between age and skeletal-cutaneous class (0.410) (Table 4).

Strong correlations were found between age and the angle between Go-Or to Po-Or (0.965) and the angle between Go-Or to normal to OP (0.397). A slightly weaker correlation was observed between age and skeletal-cutaneous class (0.410).

## 4. Discussion

The study by Bakke et al. asserts that a defect or excess on one side of the skull can lead to an imbalance of muscular activity, which may worsen with growth. Thus, one of the influencing factors is occlusion. If the occlusion is correct, an increased neuromuscular response during muscle activity has been observed [27].

Patients with crossbite are asymmetrical in both static and dynamic phases of the activity of masticatory muscles. Some authors have stated that asymmetry at rest, during maximal clenching, and during mastication is not statistically significant [23,28,29,30,31,32]. Farronato et al. proposed to investigate changes in temporalis and masseter muscle activity before and after SARPE (Surgically Assisted Rapid Palatal Expansion) in adult patients by measuring electromyographic and electrokinetic activity [33].

The biomechanical model should use cephalometric estimates of the muscle’s line of action, which connects the midpoints of two skeletal attachments, i.e., close to the muscle’s central axis. Unfortunately, muscle is a complex three-dimensional structure, and a two-dimensional representation by X-ray landmarks is only an approximation [34]. In fact, all reported analyses use Go as the posterior end of the MM surface [4,5,8,9]. This landmark is considered to be the midpoint of the mandibular attachment zone of the MM. Conversely, the midpoint of the Go and antegonion used by Osborn and Gionhaku and Lowe seemed too anterior for a human muscle [10]. The superior end of the muscle should be between the zygomatic-temporal suture and the anterior end of the maxillary process of the zygomatic bone or, if well developed, the lateral corner of the zygomatic process of the maxilla [35]. Unfortunately, identification of the corresponding cephalometric profile is often difficult, and alternative approaches have been devised.

The MM line of action used in this study was assumed to follow the line drawn between landmarks Go and Or and was derived from a study by Throckmorton and Dean [6]. This proposal appeared to be the simplest of several biomechanical models that define cephalometric landmarks that are difficult to identify on standard radiographs and thus may be of limited value in orthodontics, where X-ray exposure in pediatric patients should be limited. The angle between the line of action of the masseter muscle and the normal of the occlusal plane averaged about 40° for the patient sample and about 39° for the Bolton tracings, and the hypothesized line of action was far from perpendicular to the occlusal plane in both the orthodontic sample and in the “reference” group. In fact, it should be mentioned that the minimum joint load during symmetrical molar occlusion is predicted when the MM is 70° to 75° to the occlusal plane, i.e., 15° to 20° inclined to the normal of the occlusal plane [34]. The variation within groups is small, and the present calculations are considered to be an approximation of reality, since the lines of action of the muscles are estimated with systematic errors.

A slight decrease with aging was observed in the mean values, which can be explained by changes in both the occlusal plane (second and third molar eruption, incisor movement) and gingival angle. Considering only the adult group (patients and Bolton’s occlusion), the mean value of 37° compares well with the 33° for the long head type and 38° for the short head type found by Iwasaki in the dry cranium [12].

The values reported by Throckmorton ranged from 0° to 31° [4,6,7,8]. Osborn found an almost 1:1 relationship between the MM angle and the occlusal angle of the molars in dry skulls, which means that the muscle line is approximately parallel to the normal of the occlusal plane [10]. Gionhaku and Lowe found an average radiographic inclination angle of 21° with respect to the occlusal plane in MM and an average angle of 28° in their own study of 14 adult male subjects [14]. In both cases, the subjects were between 4 and 6 years of age. The mean values of 12° and 20° are taken from the relevant autoptic [8] and cephalometric [9] literature, respectively (see references here). In a group of young adults (22–48 years), magnetic resonance studies showed a mean inclination angle of 16°, but with large individual differences, with a maximum value of 27° [12]. However, in no case were the muscle lines defined in the same way. Furthermore, it has already been suggested that there may not be a constant relationship between the MM angle and the occlusal plane [12].

The angulation of the MM action line relative to the Frankfort plane was also calculated. In this case, the relationship with age was stronger, with correlation coefficients of 0.449 for patients (Table 2) and 0.965 for bolt tracings (Table 4). In the adult group of the orthodontic patient sample, the mean angle of 45° was 4° to 5° greater than in the pediatric group (mean 41°) and about 3° greater than in the adolescent group (mean 42°). Similar differences were found in the Bolton traces (Table 3). Thus, the present mean angle is in good agreement with the self-viewing findings of 45° by van Eijden et al. and about 54° by Kasai et al. Conversely, it differs from the 60–90° range (mean 70–78°) reported in recent magnetic resonance studies [13,14,15,17,33,34,35,36]. In magnetic resonance testing, muscles are virtually sectioned along several spatial planes and their lines of action are mathematically reconstructed in three spatial dimensions. Furthermore, individual differences in muscle position and angle have been reported [14].

The inclination of the Go-Or line relative to the Frankfort plane was not related to the anteroposterior relationship of the jaw as assessed by the skeletal-cutaneous classes (A′ and B′) projected to the occlusal plane, nor was it poorly related to the inclination of the same Go-Or line relative to the normal to the occlusal plane [22]. Conversely, there was a higher correlation between the estimated inclination of the muscle relative to the normal to the occlusal plane and the same skeletal-cutaneous class (Table 2 and Table 4). Because Kasai et al. did not analyze the jaw-jaw relationship, we could not find any literature data on this point. Kasai et al. found a significant correlation of r = 0.63 between the inclination of the masseter muscle to the occlusal plane and the saddle-nose line [13].

In this study, we studied both a standard group of well-known cephalometric patients (Bolton tracings 14) and a large, heterogeneous group of orthodontic patients of both sexes in a wide age range. No selection criteria were used for orthodontic patients, and several types of malocclusions were sampled, as indicated by skeletal-cutaneous class values. Even if the mean value of 4 mm is representative of skeletal-skin Class I, the wide range indicates that the present results are not limited to a specific subject but can be extended to the general orthodontic population [22,36,37,38].

The skeletal-cutaneous class is a measure of anterior-posterior jaw discrepancy, expressed as the linear distance between the soft tissue A′ and soft tissue B′ projections on the occlusal plane. The study found a wide range of values, from −14.5 to 15.5 mm, indicating significant individual variation in this parameter. Notably, abnormal values were observed in two adolescents, with no clear gender- or age-related trends. This suggests that anterior-posterior jaw relationships can vary greatly within the population, and such variations may not necessarily correspond to age or gender.

The average angle between the estimated line of action of the MM (Go-Or) and the normal of the occlusal plane was found to be 39.3°. This angle also displayed age-related changes, decreasing by approximately 3° in adults compared to children. However, no significant gender differences were noted. This observation implies that as individuals grow and develop, there are changes in the inclination of the MM relative to the occlusal plane. These changes could have implications for bite force and muscle function.

The angle between Go-Or and the Frankfort plane (Po-Or) had an average value of 41.99°, ranging from 30.4° to 53.8°. Interestingly, this angle displayed a more pronounced age-related pattern, increasing by an average of about 4° between ages 6 and 50. This suggests that the inclination of the MM in relation to the Frankfort plane evolves significantly with age. The correlation analysis showed that this was the most substantial age-related effect observed in the sample, highlighting the importance of considering this angle when assessing craniofacial biomechanics.

Furthermore, the similarity between the results obtained in patients and the Bolton standard, which should represent average normal craniofacial growth, suggests that the present results may be extrapolated beyond the orthodontic population. A more accurate analysis would require studying a new group of normal individuals. This is because the Bolton traces are from a population with a different ethnic origin (North American Caucasians with Northern European ancestry), a population that predates the current orthodontic population (data collection began in the 1930s). Unfortunately, there is no longer a general population outside of patients for whom invasive radiographic analysis is available, and both magnetic resonance imaging and autoptic studies are limited to small samples. Thus, biomechanical analysis must rely on either selected healthy subjects (usually adults), a small number of potentially unhealthy cadavers, or data collected from many patients of almost any age.

Studying mandibular protrusion treatment using 3D CT in rats, we found that posterior displacement in growing rats leads to a smaller mandible in adulthood [24]. Another study compared automatic cephalometric analysis using deep learning with manual tracing and found high reliability for all measurements, with only a few statistically significant differences [39,40].

The results of this study provide valuable clinical insights and potential benefits in orthodontics that follow the principles of personalized medicine. Firstly, the assessment of anterior-posterior jaw discrepancies, represented by the soft tissue Wits, across a diverse sample revealed that this parameter varied significantly among patients. Notably, two adolescents demonstrated abnormal values, highlighting the clinical importance of individualized treatment planning. Additionally, the study found that the angle between the estimated line of action of the masseter muscle and the occlusal plane showed age-related variations, which can guide orthodontic interventions tailored to different age groups. Furthermore, the correlation analysis between the anteroposterior relationship between the maxilla and mandible and the angle between the masseter muscle line of action and the occlusal plane provides orthodontists with insights into biomechanical factors influencing treatment outcomes. The data from Bolton tracings further corroborated these findings and demonstrated strong age-related correlations, reinforcing the clinical relevance of these parameters. All these findings offer orthodontic practitioners a better understanding of individualized treatment needs, age-specific considerations, and biomechanical factors, which can ultimately lead to more effective and patient-tailored orthodontic care.

The use of Cone Beam Computed Tomography (CBCT) for lateral cephalometric analysis offers distinct advantages, primarily in providing three-dimensional imaging of the craniofacial complex, allowing for more accurate and comprehensive assessments of dental and skeletal relationships. It enables orthodontists to view anatomical structures from multiple perspectives, enhancing the precision of treatment planning and monitoring. However, there are limitations associated with CBCT in lateral cephalometric analysis. Firstly, the increased radiation exposure compared to traditional two-dimensional radiography raises concerns, especially in pediatric and adolescent patients who are more susceptible to radiation’s harmful effects. Additionally, the cost and availability of CBCT machines may pose practical constraints for some dental practices. Furthermore, the extensive data generated by CBCT scans can complicate data analysis and require specialized software and training. Finally, while CBCT provides valuable 3D information, its use for routine lateral cephalometric analysis may not always be justified, as it may not significantly alter treatment decisions in straightforward cases. Therefore, the clinical decision to employ CBCT for lateral cephalometric analysis should be made judiciously, considering the specific clinical needs and limitations.

Other limitations include, the absence of longitudinal data restricts insights into how cephalometric parameters change over time within individuals. Gender-specific differences might have been missed due to sample size limitations and external validation through clinical outcomes or further experiments would enhance the study’s clinical relevance and applicability.

## 5. Conclusions

In conclusion, the current lines used to approximate MM inclination in cephalometric radiographs were readily identifiable in all cases. Its position with respect to the dental and skeletal reference (occlusal and Frankfort planes) partially agreed with the literature findings, even if different approximations of the MM line of action were made. With respect to the inclination to the Po-Or line, a significant effect of age was observed, which may explain some of the literature differences. Overall, given the important and complex relationship between craniofacial morphology and MM dimensions and inclination, the present cephalometric analysis can be usefully used to estimate the mechanical advantage of MM in biomechanical simulations of masticatory muscle performance.

### Future Directions of Research

Future research directions in the field of craniofacial biomechanics should encompass a wide range of investigations to further advance our understanding of craniofacial development, function, and clinical applications. *Longitudinal studies* tracking craniofacial changes from childhood to adulthood will provide insights into the dynamic nature of craniofacial growth. Researchers should explore the multifaceted interactions among genetic, environmental, and functional factors to capture the complexity of craniofacial morphology. Investigating potential gender disparities in craniofacial development may reveal subtle distinctions in how males and females evolve differently. To bridge the gap between research and practical application, studies should validate the utility of craniofacial parameters in clinical contexts like orthodontics and craniofacial surgery. *Leveraging advanced imaging techniques and incorporating 3D imaging and MRI* can enhance the precision of data collection. Moreover, exploring the application of research findings in the diagnosis and treatment of craniofacial disorders, such as temporomandibular joint disorders, is essential for improved patient care. Cross-population comparisons can uncover variations in craniofacial development influenced by genetics, environment, and culture. Finally, interdisciplinary collaboration among orthodontists, biomechanics experts, and anatomists can provide a holistic understanding of craniofacial complexity, ultimately benefiting both research and clinical practice.

## Figures and Tables

**Table 1:** Number of analyzed cephalograms, and descriptive statistics (mean and standard deviation in brackets) of the measured variables. Age was rounded to the nearest 6 months.

	Males	Females	All Subjects	6–10 Years	11–15 Years	16–50 Years
Orthodontic patients	217	293	510	257	134	119
Skeletal-cutaneous class (mm)	4.7 (4.1)	3.4 (3.7)	4.2 (4.0)	3.8 (3.4)	4.8 (4.2)	3.4 (5.8)
Go-Or to normal to OP (°)	39.59 (0.27)	39.12 (0.27)	39.33 (0.19)	39.92 (0.23)	39.41 (0.37)	36.87 (0.59)
Go-Or to Po-Or (°)	41.99 (0.28)	41.98 (0.22)	41.99 (0.18)	40.95 (0.19)	42.34 (0.26)	45.39 (0.50)

Skeletal-cutaneous class = linear distance between the projections of A′ and B′ points on the occlusal plane.

**Table 2:** Linear correlation coefficients between the analyzed variables in the orthodontic sample.

	Age	Skeletal-Cutaneous Class	Go-Or to Po-Or
Skeletal-cutaneous class	0.025	-	-
Go-Or to normal to OP	0.255	0.444	0.322
Go-Or to Po-Or	0.449	0.057	-

All analyses are significant at the 0.001 level.

**Table 3:** Descriptive statistics (mean and standard deviation in brackets) of the measured variables in the Bolton tracings between 6 and 18 years of age.

	All Tracings	6–10 Years	11–15 Years	16–18 Years
Skeletal-cutaneous class (mm)	3.5 (1.2)	2.8 (1.5)	4.2 (0.5)	3.8 (0.7)
Go-Or to normal to OP (°)	38.77 (0.47)	39.04 (1.11)	39.29 (0.20)	37.39 (0.45)
Go-Or to Po-Or (°)	40.72 (0.55)	38.89 (0.49)	41.16 (0.51)	41.09 (0.56)

Skeletal-cutaneous class = linear distance between the projections of A′ and B′ points on the occlusal plane.

**Table 4:** Linear correlation coefficients between the analyzed variables in the Bolton tracings.

	Age	Skeleto-Cutaneous Class	Go-Or to Po-Or
Skeleto-cutaneous class	0.410	-	-
Go-Or to normal to OP	0.397	0.530	0.481
Go-Or to Po-Or	0.965	0.367	-

All analyses are significant at the 0.001 level.

## Data Availability

Data supporting reported results can be found on the database of the Dolphin software: https://www.dolphinimaging.com/product/Imaging?Subcategory_OS_Safe_Name=Ceph_Tracing, accessed on 1 September 2023.
